# Abnormal Reorganization of Functional Cortical Small-World Networks in Focal Hand Dystonia

**DOI:** 10.1371/journal.pone.0028682

**Published:** 2011-12-13

**Authors:** Seung-Hyun Jin, Peter Lin, Mark Hallett

**Affiliations:** 1 Human Motor Control Section, National Institute of Neurological Disorders and Stroke, National Institutes of Health, Bethesda, Maryland, United States of America; 2 Department of Neurosurgery, Seoul National University Hospital, Seoul, Korea; Centre Hospitalier Universitaire Vaudois (CHUV), Switzerland

## Abstract

We investigated the large-scale functional cortical connectivity network in focal hand dystonia (FHD) patients using graph theoretic measures to assess efficiency. High-resolution EEGs were recorded in 15 FHD patients and 15 healthy volunteers at rest and during a simple sequential finger tapping task. Mutual information (*MI*) values of wavelet coefficients were estimated to create an association matrix between EEG electrodes, and to produce a series of adjacency matrices or graphs, *G*, by thresholding with network cost. Efficiency measures of small-world networks were assessed. As a result, we found that FHD patients have economical small-world properties in their brain functional networks in the alpha and beta bands. During a motor task, in the beta band network, FHD patients have decreased efficiency of small-world networks, whereas healthy volunteers increase efficiency. Reduced efficient beta band network in FHD patients during the task was consistently observed in global efficiency, cost-efficiency, and maximum cost-efficiency. This suggests that the beta band functional cortical network of FHD patients is reorganized even during a task that does not induce dystonic symptoms, representing a loss of long-range communication and abnormal functional integration in large-scale brain functional cortical networks. Moreover, negative correlations between efficiency measures and duration of disease were found, indicating that the longer duration of disease, the less efficient the beta band network in FHD patients. In regional efficiency analysis, FHD patients at rest have high regional efficiency at supplementary motor cortex (SMA) compared with healthy volunteers; however, it is diminished during the motor task, possibly reflecting abnormal inhibition in FHD patients. The present study provides the first evidence with graph theory for abnormal reconfiguration of brain functional networks in FHD during motor task.

## Introduction

Patients with focal hand dystonia (FHD) have task-specific uncontrolled muscle activity characterized by co-contraction of agonist and antagonist muscles. In terms of pathophysiology of dystonia, evidence for abnormalities of cortical dysfunction include abnormal levels of activity in sensorimotor cortex, supplementary motor cortex (SMA) and premotor cortices during motor tasks and abnormal sensorimotor processing [1]. There is a large body of evidence for abnormalities in somatosensory processing as well [2], [3]. Abnormal sensorimotor networks have been hypothesized to be the pathophysiological mechanism of distorted movement functioning. Several neurophysiologic studies have supported the finding of sensorimotor integration abnormalities in FHD using supplementary motor cortex transcortical magnetic stimulation (TMS) [4], [5] and positron emission tomography (PET) [6]. Collectively, these findings demonstrate abnormalities in sensorimotor integration in the cortex [1], [7]. However, analysis of the cortical networks involved in dystonia has mainly focused on the changes in select cortical areas such as the motor/premotor and somatosensory cortex rather than the topological organization of the large-scale functional cortical networks. Recently, we have [8] reported that FHD patients showed reduced mutual information (MI) compared to healthy volunteers in the beta band, and this allows us to look at the large-scale functional connectivity in FHD patients for the first time. Here, we investigate the functional connectivity in FHD patients more precisely, taking advantage of the recent enormous progress of the field of complex brain network analysis.

Considering that the brain is a large-scale network consisting of millions of neuronal elements that are interconnected in characteristic patterns, analysis of the interactions between various cortical areas may be essential in understanding a brain function. The human brain is perhaps the most complex entity known to science. Since any complex system in nature can be modeled as a network where nodes are the elements of the system and edges represent the interactions between them [9], the human brain also can be modeled as a network. The application of network analysis based on graph theory to diverse human brain signals such as functional magnetic resonance imaging (fMRI), magnetoencephalography (MEG), and electroencephalography (EEG) provides a complex systems approach to the study of brain functional networks [10], [11], [12].

Network architecture is regarded as a key substrate for sensorimotor and cognitive processing, which may be localized discretely in specialized regions and represented by integration through long-range communication between synchronized oscillatory neuronal assemblies for optimal information processing [13], [14]. Particularly, small-world networks, indicating high levels of clustering and short path lengths, offer a structural substrate for functional segregation and integration of the brain [15]. A network can be assessed in terms of its efficiency, which provides a vital measure of how well information is transferred over the network [9], [16]. Global (*Eglob*) and local (*Elocal*) efficiencies of small-world networks have been employed to investigate functional cortical networks in several clinical applications [17], [18], [19], [20]. Since efficiency is defined as the harmonic mean of the shortest path lengths linking two nodes, increase of *Eglob* indicates a topology of a functional network that has short path length, which should aid functional integration. Although there is some argument about the usage of *Eglob* [21], it has been reported to be superior as a measure of functional integration [16] and has been used in previous studies [17], [18], [19], [20]. Likewise, increase of *Elocal* represents increase of clustering as an index of functional segregation. In addition, Achard and Bullmore [16] suggested that the cost-efficiency (*CE*) measure is a useful concept to describe economical small-world properties combining high efficiency with low connection cost of brain networks, which is further supported by a study of cognitive function [22]. In the present study, we hypothesized that the economical small-world properties of functional cortical networks would be disrupted in FHD patients.

To investigate the hypothesis, based on the fact that neural communication depends on the process of transmitting information mediated by synchronization of neural oscillations [14], frequency band specific functional networks were derived from EEG data in healthy volunteers and FHD patients during both rest and simple finger tapping task that did not induce dystonic symptoms. Mutual information (*MI*) including linear and nonlinear connectivity was used in order to make an association matrix representing relation between each pair of nodes (here, EEG electrodes), since it is suited to measure changes in synchronization of different neuronal electrical activities [23], [24]. *MI* matrices were thresholded to generate a series of undirected and unweighted graphs and the topological properties of the networks were evaluated by graph theoretic approaches.

## Materials and Methods

### Ethics statement

The study protocol was approved by the Institutional Review Board at National Institute of Neurological Disorders and Stroke (NINDS), and all subjects gave written informed consent.

### Subjects

The study involved 15 right hand affected patients (mean age of 51.4 years; 11 males) and 15 healthy control subjects (mean age of 45.93 years; 12 males). No difference between subjects in age was found (t_28_ = 1.302, *p* = 0.204, two-sample *t*-test). All subjects were right handed. Six patients were diagnosed with musician's cramp and the others with writer's cramp; we believe these entities to be two manifestations of the same disorder. The duration of disease ranged from 2 to 25 years. For patients treated with injections of botulinum toxin (BTX) the last injections were always more than 3 months prior to testing. FHD patients and healthy volunteers were recruited from the NINDS Clinics. Clinical details of FHD patients are described in Table 1. Some of the same data were already reported in a manuscript dealing with functional connectivity in FHD patients [8] and, the normal data only, in another manuscript dealing with functional network analysis [25].

**Table 1:** Demographic and clinical details of FHD patients.

no.	Gender	Age	Type of FHD	Duration (years)	Treatment
1	Male	29	M	2	None
2	Male	54	M	11	BTX
3	Male	45	M	12	BTX
4	Male	55	M	3	None
5	Male	56	M	5	BTX
6	Male	48	M	6	BTX
7	Female	56	W	18	BTX
8	Female	50	W	10	BTX
9	Male	40	W	19	BTX
10	Female	64	W	19	BTX
11	Male	57	W	17	None
12	Male	57	W	4	None
13	Male	59	W	24	BTX
14	Male	44	W	25	None
15	Female	57	W	3	None

For patients treated with injections of botulinum toxin (BTX) the last injections were in all cases more than 3 months prior to testing.

Types of FHD: M; musician's cramp, W; writer's cramp.

### EEG recording and Preprocessing

EEG signals were recorded from 58 surface electrodes mounted on a cap (Electro-Cap International, Inc., Eaton, OH, USA) using the international 10–20 system referenced to the right earlobe electrode (A2). The left earlobe electrode (A1) was recorded as a separate channel, and we converted the EEG signals into the digitally linked earlobe reference before further analysis to reliably estimate the scalp EEG potential [26]. Bipolar recordings of the vertical and horizontal electrooculogram (EOG) and surface electromyogram from extensor digitorum communis and the first dorsal interosseus muscles were simultaneously recorded. Signals from all channels were amplified (Neuroscan Inc., El Paso, TX, USA), filtered (DC-100 Hz) and digitized with sampling frequency 1 kHz.

Resting state EEGs were recorded for 5 minutes with eyes open. For the simple sequential finger tapping task, subjects were asked to press the button of a commercial keypad (Neuroscan Inc., El Paso, TX, USA) with their right hand paced by a metronome beat at 2 Hz for 5 minutes. The sequence performed was 2-3-4-5 with each digit corresponding to index, middle, ring and little finger, respectively. After recording, we extracted 120 trials of the 4-item sequence equivalent to a total 480 key presses to assess behavioral performance. The exact sequence of key presses was considered the accuracy. Subjects were instructed to maintain their eyes open and to fixate on a target 3 m away during the entire recording to avoid extraneous movements or eye blinks and giving them any visual feedback to their performance. None of the patients became symptomatic or experienced any discomfort during a task.

Linear trend was removed from the entire epoch and eye movement related artifacts were removed using an auto-regressive exogenous input (ARX) model [27]. Finally, 5 artifact-free epochs (each epoch corresponded to 10 sec) in each subject were obtained to calculate wavelet coefficients and eventually evaluate *MI* values between wavelet coefficients for each pair of nodes. EEG was down-sampled to 500 Hz before applying Morlet wavelet transformation. The preprocessing steps were performed by using the same home-made MATLAB (MathWorks, Natick, MA) scripts as used in Bai et al. [28].

### Graph construction

We estimated *MI* values of wavelet coefficients to create an association matrix between EEG electrodes. Wavelet coefficients of the alpha (7.97∼15.05 Hz), beta (15.05∼31.25 Hz), and gamma (31.25∼50.78 Hz) frequency bands were obtained from Morlet wavelet transformation. A detailed explanation is presented in the Supplementary material (Text S1).

To yield a series of adjacency matrices or graphs *G*, the *MI* association matrix was thresholded by network cost since it concisely couples with network efficiency, thus providing a biologically meaningful description of the performance of a network [9], [16], [17], [19], [22], [25].

The degree of each node is defined as the number of edges connecting it to the rest of the graph. The degree of connectivity of a graph is the average of the degrees of all the nodes in the graph. Since the maximum possible number of edges of *N* number of nodes is 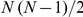, a connection density or network cost *C* can be defined as 
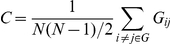
, where 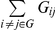 corresponds to the total number of edges in the adjacency matrix *G*. An unweighted graph *G* consisting of 58 nodes and undirected edges between nodes was constructed by applying cost, *C*, to the *MI* association matrix of wavelet coefficients of each subject at each condition. Element of *G* is given by 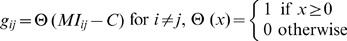, where 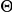 is the Heaviside function, defined as 1 if 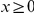, and otherwise 0.

Resulting networks depend on the choice of the threshold value, cost *C*; that is, high costs yield sparse adjacency matrices and low costs yield densely connected graphs. Here, we thresholded all *MI* matrices from the cost of 0.16, to construct a sparse graph with the mean degree *k* = 2log(*N*) = 2log(58)≈9 and total number of edges 264, equivalent to cost *C*∼0.16 or 16% of the maximum number of edges possible in a network of 58 nodes similar to the method used in previous studies [13], [16], [18], [25]. A small-world regime was determined by the criteria that small-world properties of the brain networks are determined by *Eglob* greater than a comparable regular (but less than a random graph) and *Elocal* greater than a random graph (but less than a regular) [16]. In order to clarify whether small-worldness is achieved in small-world regime, the small-world value, called sigma, was calculated.

### Efficiency and cost-efficiency of small-world networks

Efficient behavior of small-world networks was introduced as a concept by Latora and Marchiori [9]. The main advantage of efficiency measures over conventional clustering coefficient *Cp* and shortest path length *Lp* to quantify small-world properties of a network is that it provides a single variable with a clear physical meaning, i.e., the efficiency in functional connectivity, to define the small-world behavior and also allow a precise quantitative analysis of either the disconnected or non-sparse graphs or both [9].

For each cost in the range of 0.16<*C*<1.0, *Eglob* was calculated using the following equation:
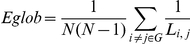



Here, *i*≠*j* denotes a node connected to *i*, and *L_i,j_* is the shortest length of the path from node *i* to node *j*. *Eglob* has been shown that it is a measure of the efficiency of a parallel information transfer, where all the nodes in the network concurrently exchange packets of information [9].

As an another global metrics, *Elocal* can be defined as
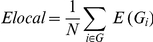
where *Gi* is the subgraph of the neighbors of a node *i*, and *E* (*Gi*) indicates the efficiency of the subgraph *Gi*. By definition the *Elocal* can be understood as a measure of the fault tolerance of the network, indicating how efficient the communication is between the first neighbors of *i* when *i* is removed [16]. This quantity can be considered as a similar measure of clustering coefficient, *Cp* (compatible with functional segregation), while *Eglob* is thought to be a similar measure of shortest path length *Lp* (compatible with functional integration) [9], [13].

Whereas *Eglob* and *Elocal* are measures of global information flow, nodal or regional efficiency, *Enodal*, was used to assess the efficiency at each node. *Enodal* can be defined as the inverse of the harmonic mean of the minimum path length between a node *i* and all other nodes in a graph [16], and it is regarded as a measure of the communication efficiency between a node *i* and the all the other nodes in the network [19]. A node with high *Enodal* will have short minimum path length to all other nodes in the graph by definition.
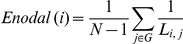
The global cost efficiency (*CE*) is defined as the difference between *Eglob* and cost, *Eglob*-*C*, which is positive in the case of an economical network [16], [22].

Comparable random and regular networks that preserved the same number of nodes and edges were synthesized to evaluate small-world properties [29]. 20 random and regular networks were generated at each cost threshold *C*. 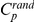 and 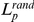 were obtained by averaging 20 populations of random networks. Since small-world networks have similar absolute path length but higher absolute clustering coefficient, small worldness index could be defined as sigma, 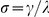 (where, 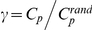, 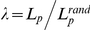), which will be greater than 1 if the network has small-world properties [30]. The clustering coefficient, path length, and efficiency estimates were obtained with functions from the Brain Connectivity Toolbox (http://www.brain-connectivity-toolbox.net/).

### Statistical analysis

Normality of the small-world parameter was graphically assessed by plotting. If the data are normal, the plot will be linear. The data had a normal distribution over the selected cost range (see Figure S1). Two-sample *t*-test was performed to detect statistical differences in the error rate as a behavioral performance between groups.

We compared the global metrics (*Eglob*, *Elocal*, *CE*, and max*CE*) at each cost value to evaluate the small-world topological differences between groups and conditions using a two-way repeated measures analysis of variance (ANOVA) with main effect of Group as a within-subject factor (two level: rest and task) and Condition as a between-subject factor (two level: FHD patients and healthy volunteers) and interaction (Group×Condition). Post-hoc *t*-tests were also conducted after performing ANONA. *P* values<0.05 were considered significant.

For the degree and *Enodal* distribution (at a cost of 0.28), the map of the connectivity was standardized by converting to *Z* scores for the group and condition separately before group averaging so that maps across participants could be averaged and compared. The conversion of *Z* score does not affect the topography of the individual-participant maps but cause the values in each participant's map to be comparably scaled [31]. Statistical tests performed on each node were reported for 3 levels, i.e., uncorrected *p*<0.05, false-positive correction (FDC) *p*<1/N = 0.017, and false discovery rate (FDR) correction *p*<0.05/N = 0.00086 [22]. The scalp plotting program used in the present study was adapted for the current use from Delorme et al. [32] Headplot Matlab script.

Pearson's correlation coefficients were evaluated to investigate the relationship between the economical small-world properties of the brain functional networks and the duration of disease as a clinical variable. All statistical analysis was performed using Statistics Toolbox in MATLAB. Data are presented as mean ± SEM.

## Results

### Behavioral results

As described in our previous paper [8], there is no significant difference in behavioral results between FHD patients and healthy volunteers (F_1, 28_ = 0.01. *p* = 0.925). A detailed box-plot is shown in Figure S2.

### Global efficiencies of small-world networks: *Eglob*, *Elocal*, and global *CE*

**Figure 1:**
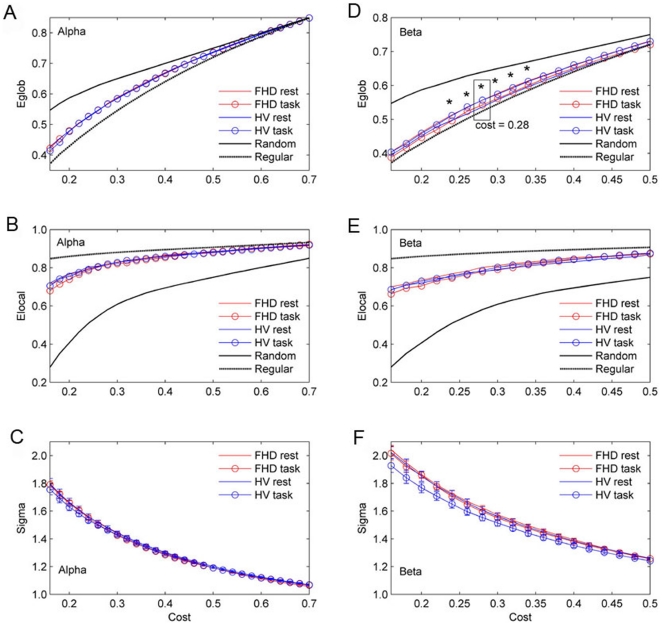
*Eglob* (a, d) and *Elocal* (b, e) as a function of cost for the random, regular, and brain networks. Small-world regime can be defined as the range of costs 0.16<*C*<0.7 in the alpha band network and 0.16<*C*<0.5 in the beta band network for which *Eglob* curve for both groups is greater than *Eglob* curve for the regular and less than random networks. Note that *Eglob* in the beta band network show interaction effect of group and condition in the range of 0.24<*C*<0.34. At a cost of 0.28, the most significant interaction of ANOVA is revealed (F_1, 56_ = 6.19, *p* = 0.016). Small-worldness is achieved within this range, since sigma is >1 in the range of costs 0.16<*C*<0.7 in the alpha network (c) and 0.16<*C*<0.5 in the beta band network (f).

Figure 1 presents the *Eglob*, *Elocal* and sigma as a function of cost for the random, regular, and brain networks. For all networks, the *Eglob* (1a, 1d) and *Elocal* (1b, 1e) increase with cost. The random graph has greater *Eglob* than the regular graph, and the regular graph has greater *Elocal* than the random graph. On average, over all subjects in each group, the brain networks of healthy volunteers and FHD patients have efficiency curves located between the limiting cases of random and regular topology. The small-world regime could be conservatively defined as the range of costs 0.16<*C*<0.7 in the alpha band networks and 0.16<*C*<0.5 in the beta band networks for which *Eglob* curve for both groups is greater than *Eglob* curve for the regular and less than random networks. The sigma plot (Fig. 1c, 1f) for the alpha and beta band networks clarify that small worldness is achieved in the small-world regime, since sigma is >1 for networks with a small-world organization [30]. *Eglob* in the beta band network shows interaction effects of group and condition in the cost range of 0.24<*C*<0.34, comprising between 24% and 34% of the maximum possible edges in a network of 58 nodes. This implies that group differences depend on the task. We found the most significant interaction of ANOVA (F_1, 56_ = 6.19, *p* = 0.016) at a cost of 0.28. For specific *F* values and *p* values, see Table 2. Since *Eglob* of the gamma band network was not located between the limiting cases of random and regular topology, we excluded it from the following analyses (see Figure S3).

**Table 2:** *Eglob* showing significant differences.

Cost, *C*	Main Effect	Healthy Volunteers		FHD		F	*p*
		Rest	Task	Rest	Task		
0.24	I	0.497±0.001	0.511±0.007	0.514±0.006	0.496±0.007	4.60	0.036
0.26	I	0.519±0.010	0.537±0.005	0.535±0.007	0.524±0.006	5.08	0.028
**0.28**	**I**	**0.537±0.009**	**0.557±0.006**	**0.557±0.007**	**0.542±0.006**	**6.19**	**0.016**
0.30	I	0.559±0.008	0.574±0.006	0.576±0.006	0.562±0.007	4.84	0.032
0.32	I	0.578±0.009	0.593±0.006	0.596±0.006	0.590±0.007	5.06	0.029
0.34	I	0.596±0.008	0.612±0.006	0.613±0.006	0.599±0.007	4.79	0.033

Two-way ANOVA was performed at each cost, and there are significant interactions in the range of 0.24<*C*<0.34. Note that at a cost of 0.28, the most significant interaction was revealed.

Mean ± SEM (standard error of mean); I, interaction.

**Figure 2:**
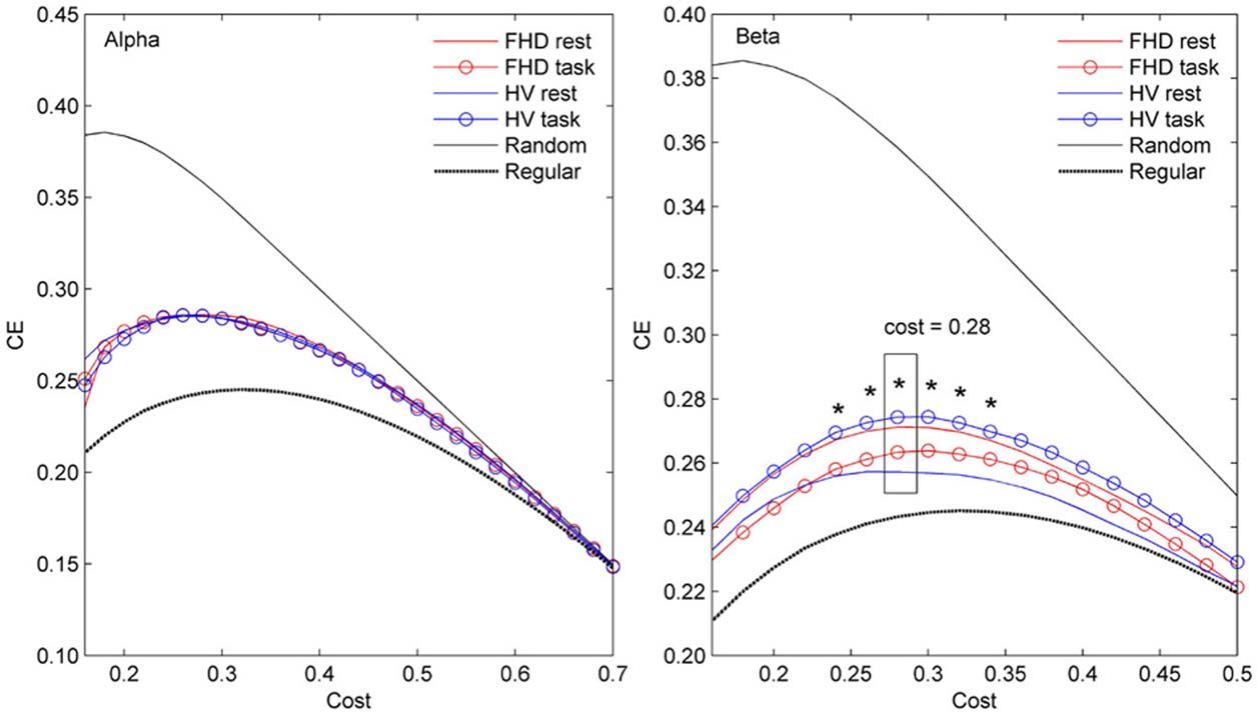
Global *CE* as a function of cost for the random, regular, and brain networks. In small world regime, 0.16<*C*<0.7 and 0.16<*C*<0.5, for the alpha and beta band networks, respectively. The *CE* curve for both groups is greater than the *CE* curve for the regular and less than for the random networks. Note that the global *CE* in the beta band network show interaction effect of group and condition in the range of 0.24<C<0.34. At a cost of 0.28, the most significant interaction was revealed (F_1, 56_ = 6.19, *p* = 0.016).

In terms of the global *CE* (Fig. 2), the alpha and beta band networks have positive values in the small-world regime, 0.16<*C*<0.7 in the alpha and 0.16<*C*<0.5 in the beta band network. This result implies that the alpha and beta band networks in both groups and conditions have economical functional networks from the *CE* standpoint, since efficiency was greater than cost. Note that the global *CE* in the beta band network shows interaction effects of group and condition in the range of 0.24<C<0.34, which is the same region as the *Eglob*, and the most significant interaction appears at a cost of 0.28.

**Figure 3:**
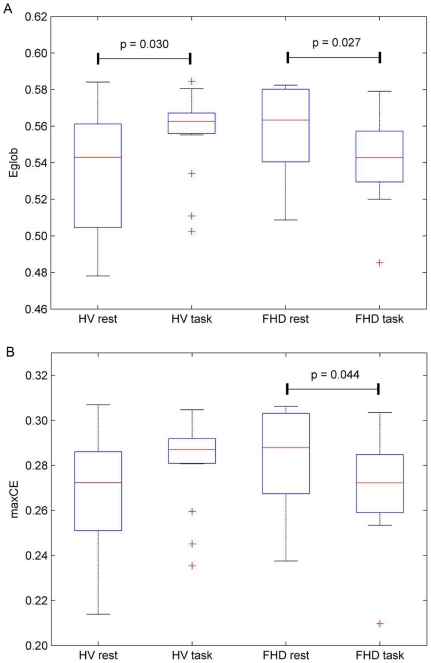
Box-plots of *Eglob* at a cost 0.28. Box-plots showing median, interquartile, and range for *Eglob* (a) at a cost 0.28, which shows the most significant interaction effects of group and condition on *Eglob*, and for max*CE* (b). *Eglob* in FHD is diminished during a task, while healthy volunteers show enhanced *Eglob*. Note that *CE* would be the same as Fig. 4a, because it denotes the *Eglob* at a given fixed cost 0.28. FHD patients show a diminished max*CE* during a task. Each horizontal line and the associated number represent the *p*-value of a post-hoc *t*-test.

Furthermore, as presented in Fig. 3a, *Eglob* at a cost of 0.28 of the beta band network in healthy volunteers was increased during the task (*p* = 0.030). In contrast, in FHD patients it was decreased during the task (*p* = 0.027). Note that *CE* would be the same as Fig. 3a, because it demonstrates the *Eglob* at a given fixed cost of 0.28. No significant difference was revealed in *Elocal* (see Figure S4). In addition, we extracted the max*CE* from *CE* curve to see whether the economical small-world property differs from each group at the maximum point summarizing the behavior of the curve [22]. Significant interaction effects of the group and condition on max*CE* were also found in the beta band (F_1, 56_ = 4.8, *p* = 0.033; Fig. 3b). FHD patients show a decreased max*CE* during a task (*p* = 0.044).

### Regional efficiency (*Enodal*) and degree distribution

Since the most significant interaction effect was detected at a cost of 0.28 in *Eglob* and *CE*, *Enodal* was tested at a cost of 0.28 to further investigate the changes of regional nodal characteristics of the functional networks. Table 3 and 4 summarize the nodes indicating significantly different *Enodal* or degree at a cost of 0.28.

**Table 3:** Nodes showing significantly different *Enodal*, at a cost of 0.28 corresponding to the maximal interaction in *Eglob*.

location	Main Effect	Healthy Volunteers		FHD		F	*p*
		Rest	Task	Rest	Task		
FCz	G	0.538±0.079	0.638±0.061	0.755±0.081	0.725±0.057	4.69	0.035
Increased *Enodal* during a task							
FC1	C	0.485±0.063	0.659±0.040	0.591±0.079	0.697±0.047	5.58	0.022
C3	C	0.490±0.053	0.689±0.046	0.462±0.071	0.656±0.063	11.05	0.0016*
C4	C	0.542±0.060	0.748±0.057	0.422±0.056	0.649±0.047	15.45	0.0002**
C3P	C	0.587±0.065	0.700±0.043	0.635±0.066	0.755±0.054	4.08	0.048
Decreased *Enodal* during a task							
POz	C	0.661±0.037	0.548±0.049	0.651±0.042	0.561±0.055	4.83	0.032
P4	C	0.719±0.040	0.586±0.078	0.791±0.035	0.697±0.031	5.29	0.025

Mean ± SEM (standard error of mean).

Main Effect; G, Group, C, Condition, I, Interaction effect.

Significant level; uncorrected *p*<0.05,

*false-positive correction (FPC) *p*<1/N = 0.017, and

**false discovery rate (FDR) correction *p*<0.05/N = 0.00086 (N = 58).

**Table 4:** Nodes showing significantly different degree at a cost of 0.28 corresponding to the maximal interaction in *Eglob*.

location	Main Effect	Healthy Volunteers		FHD		F	*p*
		Rest	Task	Rest	Task		
Increased degree during a task							
FC1	C	0.407±0.108	0.681±0.077	0.410±0.101	0.679±0.008	8.65	0.0048*
C1	C	0.875±0.104	1.126±0.072	0.752±0.109	1.109±0.060	11.7	0.0012*
C3	C	0.317±0.081	0.703±0.089	0.226±0.128	0.642±0.096	16.01	0.0002**
C4	C	0.448±0.116	0.794±0.098	0.179±0.130	0.654±0.073	14.81	0.0003**
Decreased degree during a task							
P1	C	1.297±0.066	1.172±0.068	1.381±0.083	1.211±0.055	4.62	0.0359
P5	C	0.257±0.073	0.025±0.079	0.307±0.090	0.091±0.124	5.77	0.0196
POz	C	0.939±0.067	0.690±0.071	0.897±0.067	0.721±0.090	8.44	0.0052*
PO1	C	0.791±0.094	0.659±0.078	0.854±0.052	0.658±0.080	4.53	0.0378
P4	C	0.877±0.063	0.663±0.103	0.931±0.045	0.860±0.054	4.15	0.0463

Mean ± SEM (standard error of mean).

Main Effect; G, Group, C, Condition, I, Interaction effect.

Significant level; uncorrected *p*<0.05,

*false-positive correction (FPC) *p*<1/N = 0.017, and

**false discovery rate (FDR) correction *p*<0.05/N = 0.00086 (N = 58).

**Figure 4:**
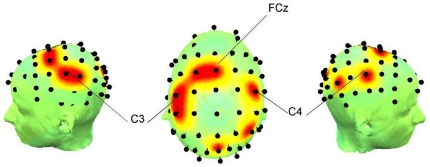
*P* maps from the left, top and right views. Each point corresponds to the location shown in Table 3, indicating significantly different *Enodal*, at a cost of 0.28 corresponding to the maximal interaction in *Eglob*. differences. At FCz channel, the main group effect was found.

**Figure 5:**
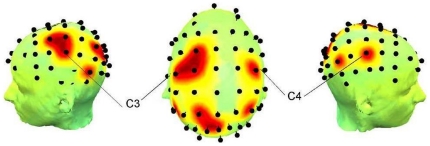
*P* maps from the left, top and right views. Each point corresponds to the location shown in Table 4, indicating significantly different degree across subjects at a cost of 0.28. C3 and C4 were the channels showing strongly enhanced degree during a task (*p*<0.00086).

Figures 4 and 5 show the *Enodal* and degree distributions, and main nodes showing significant difference across subjects.

*Enodal* and degree were significantly increased over bilateral M1, and the left sensorimotor area, while the posterior parietal areas show decreased activity during a task. The main group effect appears at FCz electrode which corresponds to the supplementary motor area (SMA). FHD patients have high *Enodal* compared with healthy volunteers at FCz. *Enodal* at FCz was diminished in FHD patients, whereas it was enhanced in healthy volunteers during the motor task.

### Relationship between efficiency metrics and duration of disease

**Figure 6:**
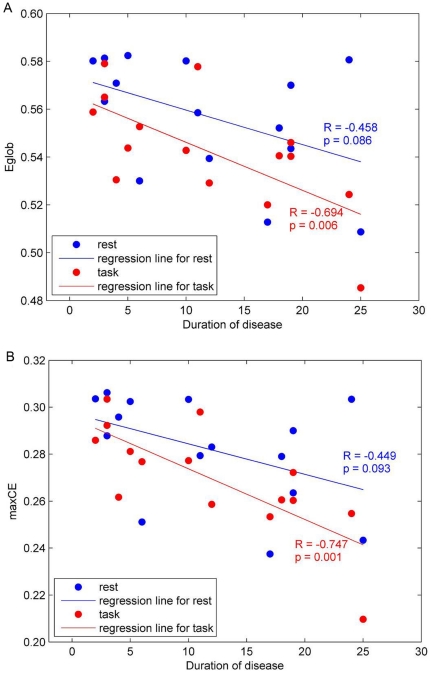
Relation between *Eglob* at a cost of 0.28 (a) and max*CE* (b), and duration of disease. Significant negative correlations are seen in both comparisons, especially during a task (red).

Figure 6a shows the correlation between *Eglob*, and duration of disease at a cost of 0.28 corresponding to the maximal interaction found in the comparison between subject groups in the *Eglob* and *CE*. A similar pattern was found in the relationship between max*CE* and duration of disease (Fig. 6b) during the task.

Table 5 displays the correlation coefficients and *p* values between *Eglob* and *Elocal*, and duration of disease at each cost in the range of 0.16<*C*<0.50 in the beta band network. We found that *Eglob* was negatively correlated with duration of disease during a task. There were also negative correlations between *Elocal* and duration of disease, even though no significant difference had been shown in the comparison with healthy volunteers (see Fig. S4). It is worthwhile to note that all the significant results show negative correlations in *Eglob*, *Elocal* and max*CE*. A longer duration of disease induced a lower global, local and maximal cost-efficiency.

**Table 5:** Correlation coefficients *R* and *p* values between *Eglob*, *Elocal* and duration of disease at each cost in the range of 0.16<*C*<0.50 in the beta band network.

Cost, *C*	*Eglob*				*Elocal*			
	Rest		Task		Rest		Task	
	*R*	*p*	*R*	*p*	*R*	*p*	*R*	*p*
0.16	−0.239	0.391	−0.419	0.120	−0.145	0.607	−0.369	0.176
0.18	−0.277	0.317	−0.347	0.205	−0.195	0.486	−0.201	0.473
0.20	−0.246	0.377	−0.392	0.149	−0.278	0.316	−0.235	0.399
0.22	−0.390	0.150	−0.420	0.119	−0.180	0.521	−0.290	0.294
0.24	−0.535	**0.040**	−0.420	0.119	−0.129	0.648	−0.331	0.228
0.26	−0.498	0.059	−0.694	**0.004**	−0.079	0.780	−0.320	0.245
0.28	−0.458	0.086	−0.672	**0.006**	−0.080	0.777	−0.234	0.400
0.30	−0.474	0.074	−0.735	**0.002**	−0.173	0.538	−0.332	0.226
0.32	−0.421	0.118	−0.763	**0.001**	−0.103	0.714	−0.516	**0.049**
0.34	−0.420	0.119	−0.812	**0.000**	−0.033	0.908	−0.740	**0.002**
0.36	−0.442	0.099	−0.729	**0.002**	−0.096	0.734	−0.753	**0.001**
0.38	−0.436	0.104	−0.726	**0.002**	−0.082	0.771	−0.615	**0.015**
0.40	−0.450	0.092	−0.735	**0.002**	−0.103	0.714	−0.477	0.073
0.42	−0.426	0.114	−0.689	**0.005**	−0.130	0.644	−0.636	**0.011**
0.44	−0.467	0.079	−0.720	**0.002**	−0.201	0.473	−0.660	**0.007**
0.46	−0.476	0.073	−0.721	**0.002**	−0.200	0.474	−0.680	**0.005**
0.48	−0.396	0.144	−0.699	**0.004**	−0.310	0.260	−0.696	**0.004**
0.50	−0.330	0.229	−0.685	**0.005**	−0.313	0.256	−0.705	**0.003**

On the other hand, no correlations between age and duration of disease, or efficiency measures including *Eglob*, *Elocal* and max*CE* were revealed (see Table S1 and S2).

## Discussion

We investigated the functional cortical networks of brain in FHD patients using graph theoretic measures to evaluate economical features. To our best knowledge, this is the first study to demonstrate the reorganization of brain functional networks in FHD patients using EEG.

### Global efficiencies of small-world networks

The economical small-world properties of the brain functional networks in healthy volunteers and FHD patients in both the rest and task conditions were found over a wide cost range of 0.16<*C*<0.7 in the alpha, and 0.16<*C*<0.5 in the beta band. This is in line with previous studies demonstrating the economical small-world properties of human brain [16], [18], [19], [22], [25] as well as their alteration due to aging and drug effects [16], or neuropsychiatric disorders such as schizophrenia [18], [22], ADHD [19] and stroke [20]. In the present study during the task, healthy volunteers showed enhanced economical small-world properties whereas the FHD patients consistently showed reduced economical small-world properties in *Eglob*, *CE* and max*CE* (Fig. 1, 2, and 3) even though no dystonic symptoms were evoked and no behavioral differences were induced. The differences in functional cortical networks appeared in the beta band network not in the alpha band.

A possible interpretation for the reduced economical small-world properties in FHD patients during a task may be explained by an abnormal network reconfiguration. In a biological context, highly evolved nervous systems are capable of rapid, real-time integration of information across segregated brain regions, which are accomplished by dynamic functional interactions in large-scale networks of the brain [33]. More directly, Bassett et al. [22] demonstrated that optimization of the emergent behavior of an information processing system can indeed depend on finding the topological configuration of the network that maximizes efficiency for minimum cost. It has been suggested that any abnormal shift caused by brain diseases toward either random [34], [35], [36] or regular [17] networks may reflect a less optimal network organization. Considering that *Eglob* is affected by the loss of long-range connections [9], and the beta band oscillations are important in long-range synchronization [37], [38], [39], abnormal beta band network reconfiguration may represent the disruption of the long-range communication among parts of the brain in FHD patients. In addition, the functional impairment associated with disorders can be theoretically related to the abnormal integration of spatially distributed brain regions that would normally constitute a large-scale network subserving function [22]. Thus, our results seem to demonstrate the abnormal functional integration of the beta band functional cortical networks in FHD patients, which is in line with our previous work [8] showing that FHD patients showed an abnormal functional integration in terms of the functional connectivity.

It has been suggested that the beta band has a specific role in movements. EEG studies found movement-related power and coherence changes in the beta frequency [40], [41], [42], and the importance of the beta band in movement control was also presented in a MEG study [43]. Thus, since changes in the motor aspects of a task are mostly reflected in the beta band [44], it seems that the beta functional network is also more sensitively reconfigured by movement.

### Relationship between efficiency metrics and duration of disease

Notably, negative correlations between efficiency measures including *Eglob*, *Elocal* and max*CE*, and duration of disease were found, indicating that the longer duration of disease, the less efficient the beta band network in FHD patients during movement. These findings demonstrate that the efficiencies in small-world structure of the beta band network in FHD patients are affected by the duration of disease. Since negative correlations between age and efficiencies in brain functional networks [16], and the influence of age on functional networks [45] have been reported, correlation coefficients between age and efficiency measures were also calculated. However, any correlations between age and efficiency measures were not revealed. Seemingly, the appearance of the less optimal network reorganization in FHD patients during a motor task is likely related to expression as well as duration of disease.

### Regional efficiency and degree distribution

As for the results of regional characteristics, the main condition effects on the *Enodal* and degree at a cost of 0.28 (Tables 3, 4 and Figs. 4, 5), showing an increase of *Enodal* and degree in primary sensorimotor regions, may be interpreted as an increased demand to support the motor command and subsequent execution. The primary sensorimotor cortex has been considered the main executive locus for simple voluntary movements [46], and Witt et al. [47] pointed out that the involvement of the region during finger tapping tasks has been consistently observed in many studies through their activation likelihood estimation meta-analysis. Parietal cortex has been shown to be active during both the execution and production of complex sequential motor tasks [48], [49], [50], [51], [52], and during auditory cued movements [53], whereas decreases of degree and *Enodal* over parietal regions were consistently observed in the present study. One explanation for this result might be due to the absence of given feedback during the performance. Visual feedback was given in previous studies that reported the activation of parietal lobe [48], [49], [50], [52], and the posterior parietal lobe may play a crucial role in the evaluation of self-generated movements [54]. However, in the present study, subjects were instructed to keep their eyes fixed during the entire recording to avoid giving them any visual feedback, so that the evaluation of self-generated movements may not have been required. Another explanation would be that there was less effort for execution of the task due to the simplicity. The involvement of parietal area was correlated with the complexity of sequential motor task [48], [51], which might require the internal visualization of finger movements in space [48] or rising levels of attention to process increasingly complex sequences [51]. However, it seems that our task was simple enough not to evoke the involvement of the parietal areas.

An important result from analysis of *Enodal* is that the main group effect was centered around SMA (FCz electrode). It has been well accepted that M1 is mainly responsible for the efferent drive of motoneurons and directly controls movements, whereas the SMA is a “higher” motor area which is involved in the preparation and initiation of movements and motor learning [43], [55]. Even simple finger movements induce functional coupling between both sensorimotor cortices and SMA [46], [56]. A dominant increase of task-related coherence between left and right lateral central electrodes (C3, C4) and medial frontocentral electrodes (FCz) was found during externally paced movements with a metronome beat of 2 Hz, which is the same condition in our study. Thus, the involvement of SMA as a locus giving the main group effect may reflect the different influence of SMA on the beta band network of FHD patients. In multiple studies, SMA activation has been observed as a neural correlate of inhibition using EEG, fMRI or both [57], [58], [59]. A hallmark of the pathophysiology of FHD has been impaired inhibitory function [1]. Although the results may vary depending on methodological differences, PET activation and fMRI studies have frequently demonstrated abnormal activity in premotor and SMA cortices in patients with dystonia [1]. Thus, we speculate that our result may reflect the high demand or effort for inhibition resulting in shorter connections from SMA, which is demonstrated by high *Enodal*. However, further study would be required in this regard, since there could be several factors affecting *Enodal* at FCz in network topology such as the global characteristics of the network. On the other hand, *Enodal* at SMA in FHD patients was diminished during the task, while healthy volunteers showed the enhanced *Enodal* at SMA. These results therefore also support the abnormal connectivity at SMA in FHD patients, which is in line with the previous studies indicating reduced activation when FHD patients performed a task that did not induce dystonic symptoms [60], [61].

### Methodological limitations

We should note that a limitation of the present study is the use of undirected and unweighted graphs. Directional information flow could be estimated by employing time delay [62], [63], and linear and nonlinear information flow can be differentiated by proper surrogate tests [64]. However, in the present study, we used zero time lag *MI*, resulting in undirected graphs, and since we did not apply surrogate tests, connections among the nodes consist of both linear and nonlinear interactions. In addition, we used unweighted graphs which may affect evaluation of the network topology, because weak and potentially non-significant links could be eliminated from the network [65]. The topology of a network might be different depending on the threshold chosen to match connection density of graph measures as van Wijk et al.[66] suggested.

Thus, further study with directed and/or weighted graphs would provide more specified network topology and characteristics of brain functional networks in FHD patients. Another limitation of the present study is that the functional networks constructed here are limited to the cortical level since we used EEG signal. Thus, in order to investigate the sub-cortical brain functional networks, neuroimaging techniques like fMRI would be required.

## Supporting Information

Figure S1 **Normality of variables was graphically assessed by plotting.** The data had a normal distribution over the selected cost range. The following figure shows the normality test results for global efficiency, *Eglob*. Since all plots are linear, the variables are regarded as having normal distribution.
(DOCX)

Figure S2 **Box-plots showing median, interquartile, and range for accuracy of 480 key presses in each group.** No significant difference was found between two groups.
(DOCX)

Figure S3 **Since the gamma networks do not satisfied with the criteria claimed by Achard and Bullmore (2007) that SW properties of the brain networks are diagnosed by** ***Eglob*** **greater than a comparable regular (but less than a random graph) and** ***Elocal*** **greater than a random graph (but less than a regular), we excluded it from our study.**
(DOCX)

Figure S4 **Box-plots showing median, interquartile, and range for** ***Elocal*** **in each group and condition at a cost 0.28, which shows the most significant interaction effects of group and condition on** ***Eglob*** **. No significant differences were found.**
(DOCX)

Table S1 **Correlation coefficients** ***R*** **and** ***p*** **values between** ***Eglob*** **,** ***Elocal*** **and age at each cost in the range of 0.16<** ***C*** **<0.50 in the beta network.** No correlations were found.
(DOCX)

Table S2 **Correlation coefficients** ***R*** **and** ***p*** **values between max** ***CE*** **and age in the beta network.** No correlations were found.
(DOCX)

Text S1 **Wavelet coefficients and mutual information estimation.**
(DOCX)
