# Supplementary material for: Abnormal Reorganization of Functional Cortical Small-World Networks in Focal Hand Dystonia
Source: PLoS One. 2011 Dec 13;6(12):e28682. doi: 10.1371/journal.pone.0028682 (PMC3236757; doi:10.1371/journal.pone.0028682)

Figure S1. Normality of variables was graphically assessed by plotting. The data had a normal distribution over the selected cost range. The following figure shows the normality test results for global efficiency, *Eglob*. Since all plots are linear, the variables are regarded as having normal distribution.


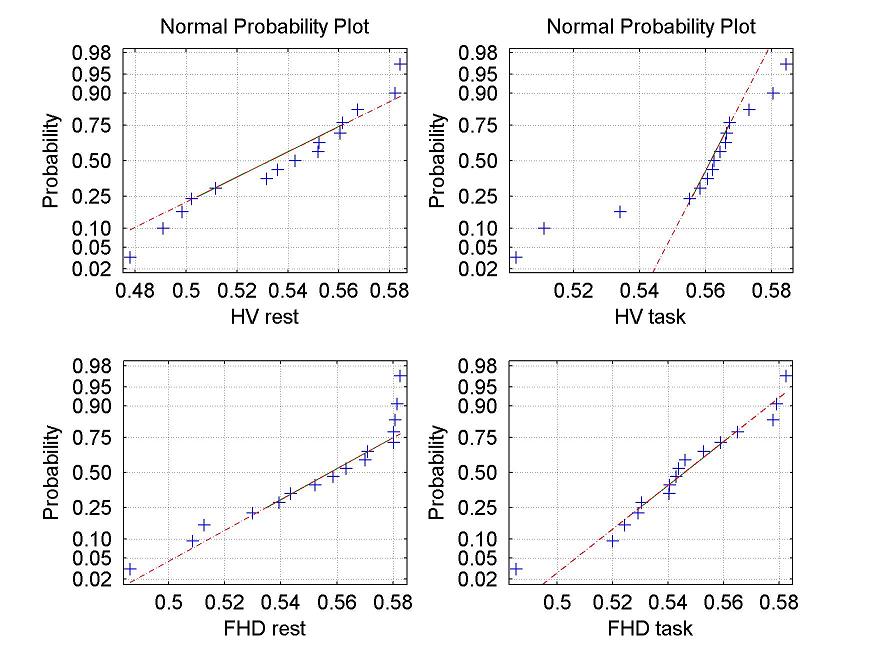

Supplement: Figure S1 — Normality of variables was graphically assessed by plotting. The data had a normal distribution over the selected cost range. The following figure shows the normality test results for global efficiency, Eglob. Since all plots are linear, the variables are regarded as having normal distribution. (DOCX) [file pone.0028682.s001.docx]
