# Supplementary material for: Abnormal Reorganization of Functional Cortical Small-World Networks in Focal Hand Dystonia
Source: PLoS One. 2011 Dec 13;6(12):e28682. doi: 10.1371/journal.pone.0028682 (PMC3236757; doi:10.1371/journal.pone.0028682)

Figure S2. Box-plots showing median, interquartile, and range for accuracy of 480 key presses in each group. No significant difference was found between two groups.


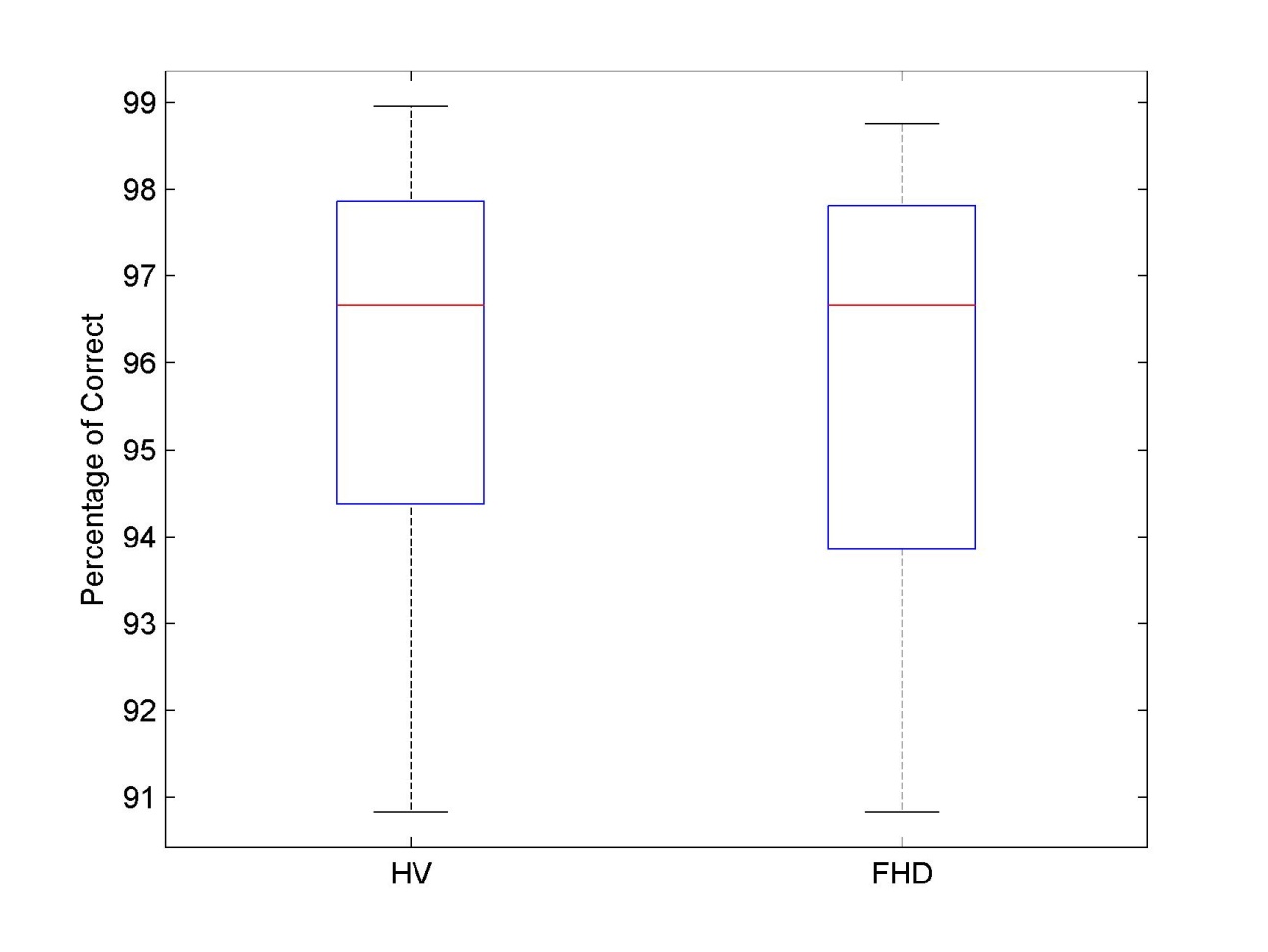

Supplement: Figure S2 — Box-plots showing median, interquartile, and range for accuracy of 480 key presses in each group. No significant difference was found between two groups. (DOCX) [file pone.0028682.s002.docx]
