# Supplementary material for: Abnormal Reorganization of Functional Cortical Small-World Networks in Focal Hand Dystonia
Source: PLoS One. 2011 Dec 13;6(12):e28682. doi: 10.1371/journal.pone.0028682 (PMC3236757; doi:10.1371/journal.pone.0028682)

Figure S3. Since the gamma networks do not satisfied with the criteria claimed by Achard and Bullmore (2007) that SW properties of the brain networks are diagnosed by *Eglob* greater than a comparable regular (but less than a random graph) and *Elocal* greater than a random graph (but less than a regular), we excluded it from our study.


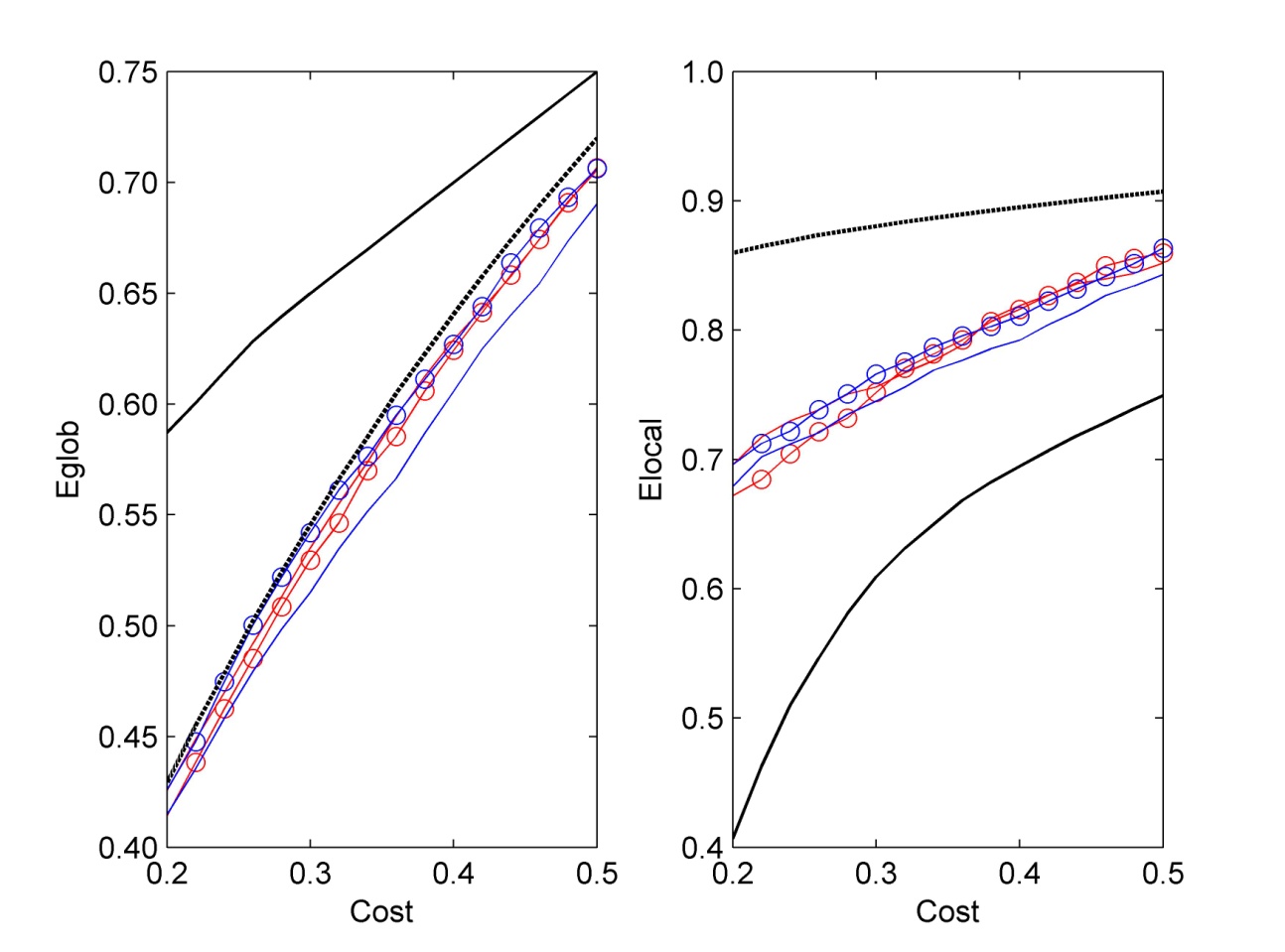

Supplement: Figure S3 — Since the gamma networks do not satisfied with the criteria claimed by Achard and Bullmore (2007) that SW properties of the brain networks are diagnosed by Eglob greater than a comparable regular (but less than a random graph) and Elocal greater than a random graph (but less than a regular), we excluded it from our study. (DOCX) [file pone.0028682.s003.docx]
