# Supplementary material for: Abnormal Reorganization of Functional Cortical Small-World Networks in Focal Hand Dystonia
Source: PLoS One. 2011 Dec 13;6(12):e28682. doi: 10.1371/journal.pone.0028682 (PMC3236757; doi:10.1371/journal.pone.0028682)

Figure S4. Box-plots showing median, interquartile, and range for *Elocal* in each group and condition at a cost 0.28, which shows the most significant interaction effects of group and condition on *Eglob*. No significant differences were found.


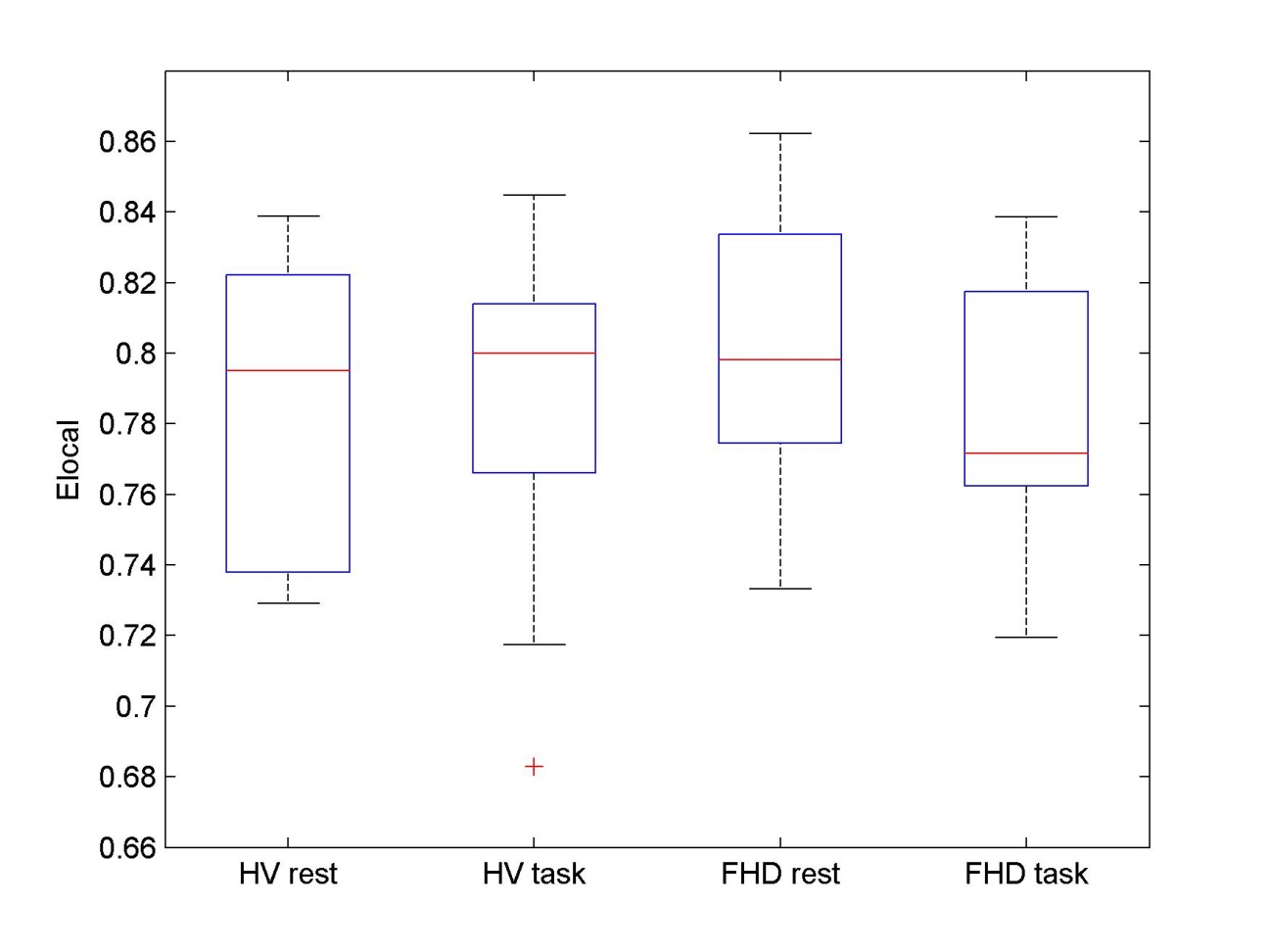

Supplement: Figure S4 — Box-plots showing median, interquartile, and range for Elocal in each group and condition at a cost 0.28, which shows the most significant interaction effects of group and condition on Eglob . No significant differences were found. (DOCX) [file pone.0028682.s004.docx]
