# Supplementary material for: Abnormal Reorganization of Functional Cortical Small-World Networks in Focal Hand Dystonia
Source: PLoS One. 2011 Dec 13;6(12):e28682. doi: 10.1371/journal.pone.0028682 (PMC3236757; doi:10.1371/journal.pone.0028682)
Supplement: Table S1 — Correlation coefficients R and p values between Eglob , Elocal and age at each cost in the range of 0.16< C <0.50 in the beta network. No correlations were found. (DOCX) [file pone.0028682.s005.docx]

Table S1. Correlation coefficients *R* and *p* values between *Eglob*, *Elocal* and age at each cost in the range of 0.16 < *C* < 0.50 in the beta network. No correlations were found.

| Cost, *C* | *Eglob* | | | | *Elocal* | | | |
| --- | --- | --- | --- | --- | --- | --- | --- | --- |
|  | Rest | | Task | | Rest | | Task | |
|  | *R* | *p* | *R* | *p* | *R* | *p* | *R* | *p* |
| 0.16 | 0.035 | 0.902 | 0.161 | 0.566 | 0.210 | 0.452 | -0.130 | 0.643 |
| 0.18 | -0.019 | 0.946 | 0.234 | 0.402 | 0.136 | 0.630 | 0.239 | 0.392 |
| 0.20 | -0.024 | 0.932 | 0.226 | 0.418 | -0.033 | 0.906 | 0.377 | 0.166 |
| 0.22 | -0.034 | 0.904 | 0.227 | 0.416 | -0.103 | 0.715 | 0.362 | 0.185 |
| 0.24 | -0.001 | 0.997 | 0.239 | 0.391 | 0.078 | 0.784 | 0.307 | 0.265 |
| 0.26 | 0.029 | 0.918 | -0.050 | 0.860 | 0.070 | 0.806 | 0.454 | 0.089 |
| 0.28 | -0.013 | 0.963 | 0.028 | 0.921 | 0.036 | 0.900 | 0.360 | 0.188 |
| 0.30 | -0.008 | 0.977 | 0.030 | 0.914 | 0.049 | 0.862 | 0.238 | 0.392 |
| 0.32 | -0.064 | 0.821 | -0.007 | 0.981 | -0.127 | 0.651 | 0.201 | 0.473 |
| 0.34 | -0.102 | 0.716 | -0.071 | 0.801 | -0.103 | 0.715 | 0.086 | 0.760 |
| 0.36 | -0.119 | 0.673 | 0.064 | 0.821 | -0.089 | 0.754 | 0.193 | 0.490 |
| 0.38 | -0.115 | 0.684 | 0.105 | 0.709 | -0.056 | 0.842 | 0.284 | 0.304 |
| 0.40 | -0.140 | 0.619 | 0.086 | 0.759 | -0.106 | 0.708 | 0.234 | 0.402 |
| 0.42 | -0.098 | 0.728 | 0.052 | 0.853 | -0.186 | 0.508 | -0.028 | 0.920 |
| 0.44 | -0.086 | 0.762 | 0.012 | 0.967 | -0.232 | 0.405 | -0.051 | 0.857 |
| 0.46 | -0.050 | 0.860 | -0.015 | 0.956 | -0.232 | 0.405 | -0.035 | 0.902 |
| 0.48 | -0.087 | 0.758 | -0.035 | 0.902 | -0.291 | 0.292 | -0.132 | 0.639 |
| 0.50 | -0.160 | 0.568 | -0.048 | 0.864 | -0.261 | 0.347 | -0.110 | 0.697 |
