# Supplementary material for: Abnormal Reorganization of Functional Cortical Small-World Networks in Focal Hand Dystonia
Source: PLoS One. 2011 Dec 13;6(12):e28682. doi: 10.1371/journal.pone.0028682 (PMC3236757; doi:10.1371/journal.pone.0028682)
Supplement: Table S2 — Correlation coefficients R and p values between max CE and age in the beta network. No correlations were found. (DOCX) [file pone.0028682.s006.docx]

Table S2. Correlation coefficients *R* and *p* values between max*CE* and age in the beta network. No correlations were found.

| Rest | | Task | |
| --- | --- | --- | --- |
| *R* | *p* | *R* | *p* |
| -0.0809 | 0.7744 | 0.0255 | 0.9282 |
