# Supplementary material for: Abnormal Reorganization of Functional Cortical Small-World Networks in Focal Hand Dystonia
Source: PLoS One. 2011 Dec 13;6(12):e28682. doi: 10.1371/journal.pone.0028682 (PMC3236757; doi:10.1371/journal.pone.0028682)
Supplement: Text S1 — Wavelet coefficients and mutual information estimation. (DOCX) [file pone.0028682.s007.docx]

**Wavelet coefficients and Mutual information estimation**

We estimated the wavelet coefficients of alpha, beta, and gamma frequency bands using Morlet wavelet transformation in order to assess the dependency of frequency contents of the cortical activity. Time-frequency representation is constructed from the continuous wavelet transform, *cwt*(*t*), using Morlet wavelet.

$cwt(t) = \int S(t) \frac{1}{\sqrt{a}} \psi\left(\frac{t-b}{a}\right) dt$,

where *ψ* is the Morlet wavelet, *a* is the scale, and *b* is the position along the time vector. Thus, for any given scale *a*, the wavelet coefficients *cwt*(*t*) are computed from *b* =1 to *b* equals the length of *S*(*t*). These computations are performed using wavelet toolbox in MATLAB (MathWorks, Natick, MA).

To create an association matrix, *MI* values of wavelet coefficients were estimated between EEG electrodes. *MI* is a measure of the amount of shared information gained about one system from the observation of another one. If two time series $X (t)$and $Y (t)$ $(t=1, ..., T)$at *T* discrete points are considered, each time series can be regarded as a random variable with underlying probability density function (PDF), $p (X (t),n)\equiv p(X(t))$with *n*=1,…,bin, the index of sampling bins for the construction of approximated PDF. In order to quantify the degree of dependence one may compute *MI* as follows using PDF:

Where, $p (X(t), Y(t) )$ is the joint PDF between $X (t)$and $Y (t)$.

*MI* is strictly non-negative and has a maximum value when the two time series are completely identical. If one system is completely independent of the other, *MI* is zero because it is assumed that 0∙log(0)=0. In this study, 32 bins were adopted for 4,096 samples. We took logarithm with base 2, so maximum *MI* in our study is 5 bits.

We added a corrective term to compensate the effect of finite data and quantization on the PDF, since the calculated entropy and *MI* have a functional dependence on the amount of data and the quantization chosen (Roulston, 1999).

where and are the number of bins for which $p (X) > 0$ and $p (Y) > 0$, is the number of bins for which $p (X, Y) > 0$, and *N* is the size of the time series. is the corrected *MI*, which is accepted as a final *MI* value.

Before applying Morlet wavelet transformation, EEG was down-sampled to 500Hz. After estimating wavelet coefficients of each frequency band, we calculated *MI* values between wavelet coefficients for each pair of electrodes, which produce an association matrix based on *MI*.

**Reference**

Roulston MS. Estimating the errors on measured entropy and mutual information. Physica D 1999;125:285-294.
